# Associations between fat-soluble vitamin status, growth, and development in Chinese children aged 3–14 years: a cross-sectional study

**DOI:** 10.3389/fped.2026.1829557

**Published:** 2026-09-11

**Authors:** Jun-Hu Zheng, Ming-Ming Li, Yi-Hua Huang, Zhi-Pin Zhang, Yi-Chong Pan, Fang-Lu Ji, Ye Wu, Hong Chen

**Affiliations:** 1 Department of Neonatology, Ruian Maternal and Child Health Hospital, Ruian, China; 2 Department of Pharmacy, Ruian Maternal and Child Health Hospital, Ruian, China; 3 Children's Health Department, Ruian Maternal and Child Health Hospital, Ruian, China; 4 Department of Laboratory, Ruian Maternal and Child Health Hospital, Ruian, China

**Keywords:** adolescents, children, cross-sectional study, fat-soluble vitamins, growth and development, nutritional status, vitamin k

## Abstract

**Background:**

The status of fat-soluble vitamins (A, D, E, K1, and K2) is crucial for childhood growth and development. However, comprehensive data on their levels—particularly vitamin K2—and their association with growth phenotypes in Chinese children remain limited. This study aimed to establish reference data and investigate these relationships.

**Methods:**

A cross-sectional study was conducted involving 1,802 children (892 boys, 910 girls) aged 3–14 years undergoing health check-ups in Ruian City, located in a subtropical monsoon climate zone in Eastern China. Serum levels of vitamins A, D, E, K1, and K2 were measured using liquid chromatography-tandem mass spectrometry. Participants were classified into healthy, short stature, obesity, or precocious puberty groups based on standardized diagnostic criteria (detailed in Methods). Differences in vitamin levels were analyzed across sex, age, season, and growth groups, and multivariate regression models were used to adjust for potential confounders.

**Results:**

Vitamin D (VD) and vitamin K2 (VK2) deficiencies were common. The overall insufficiency rates were: for VD, 13.6% in boys and 22.4% in girls; for VK2, 36.0% in boys and 39.7% in girls. Marked seasonal variations were observed: VD deficiency peaked in winter and spring, especially among girls aged 9–12 years, while VK2 deficiency was prominent in boys aged 8–9 years during spring. After adjusting for age, sex, and BMI, a significant inverse association was observed between VK status and precocious puberty in girls (*P* < 0.001). Compared with the healthy group, boys with obesity had higher vitamin A (VA) levels (potentially related to adipose tissue distribution volume), whereas girls with precocious puberty showed significantly lower levels of both VK1 and VK2 (*P* < 0.0001), leading to the hypothesis that this may be linked to accelerated bone metabolism.

**Conclusion:**

This study reveals a high prevalence of VD and VK2 deficiency among children in Eastern China, with distinct seasonal and gender patterns. In multivariate analysis, an independent inverse association was observed between VK status and precocious puberty in girls, suggesting a possible metabolic link that warrants further investigation. However, given the cross-sectional design, no causal inference can be drawn, and this finding should be regarded as hypothesis-generating. These results underscore the need for seasonally tailored, vitamin-specific nutritional attention in pediatric populations, while highlighting the importance of prospective studies to clarify the clinical significance of these associations.

## 1 Introduction

Childhood and adolescence are critical periods for physical growth, skeletal development, and immune maturation. Fat-soluble vitamins (A, D, E, and K) play indispensable roles in these processes (1, 2). Vitamin A (VA) is essential for vision, immunity, and cellular differentiation (3). Vitamin D (VD) is paramount for calcium homeostasis and bone mineralization (4). Vitamin E (VE) acts as a key antioxidant, protecting cell membranes (5). Vitamin K (VK), existing as phylloquinone (VK1, from plants) and menaquinones (VK2, from animal/fermented foods), is a cofactor for proteins involved in coagulation (VK1) and bone/vascular health (VK2, especially menaquinone-4, MK-4) (6, 7).

Despite their importance, deficiencies of these vitamins are prevalent among Chinese children (8, 9). While some national surveys have reported on VA and VD (10), population data on VK, particularly VK2, in children remain scarce and recent studies are just beginning to emerge (8). Recent technological advances in liquid chromatography-tandem mass spectrometry (LC-MS/MS) have enabled accurate measurement of these low-abundance vitamins (11). Furthermore, the relationship between comprehensive fat-soluble vitamin status and specific growth disorders (e.g., short stature, obesity, precocious puberty) is poorly characterized. Most studies focus on single vitamins, neglecting potential interrelationships, such as the well-documented synergy between VD and VK in bone metabolism (12), and their combined association with developmental phenotypes.

To address these gaps, this study aimed to: 1) assess the serum levels of five major fat-soluble vitamins (A, D, E, K1, and K2) in a cohort of children aged 3–14 years from Ruian City, China; 2) analyze their variations by sex, age, and season; and 3) explore their associations with different growth and development statuses (healthy, short stature, obesity, precocious puberty). This work provides a holistic view of fat-soluble vitamin nutrition and offers evidence for targeted interventions.

## 2 Methods

### 2.1 Study participants

A total of 1,802 children (892 boys, 910 girls) aged 3–14 years who underwent routine health examinations at Ruian Maternal and Child Health Hospital between October 2022 and February 2024 were enrolled consecutively. Ruian is located in Zhejiang Province, characterized by a subtropical monsoon climate with distinct seasons, which influences sunlight exposure and seasonal dietary patterns. Inclusion criteria were: age 3–14 years and provision of a fasting blood sample. Exclusion criteria included: known metabolic or endocrine diseases (e.g., thyroid disorders, rickets); major organ dysfunction (liver, kidney, heart); acute infection or antibiotic use within the preceding two weeks; and incomplete demographic or laboratory data. The study protocol was approved by the Ethics Committee of Ruian Maternal and Child Health Hospital (Approval No: YJ2023002). Written informed consent was obtained from all parents or guardians.

The sample size was determined based on the primary objective of estimating the prevalence of vitamin deficiencies. Assuming a VD deficiency prevalence of approximately 20% based on prior regional data (9), a sample size of at least 246 participants per group would provide 95% confidence and 5% precision. The final sample of 1,802 participants provides adequate power for subgroup analyses.

### 2.2 Classification

Participants were categorized into four groups based on clinical diagnosis and anthropometry using standardized criteria, detailed as follows:

Healthy: No identified growth or developmental abnormalities.

Short stature: Height below the 3rd percentile for age and sex, according to the “Growth Standards for Chinese Children and Adolescents” issued by Li H, et al. (13), which provides the standard growth charts for Chinese children aged 0–18 years.

Obesity: Body mass index (BMI) ≥ 95th percentile for age and sex, according to the “WS/T 586–2018 Screening for Overweight and Obesity among School-age Children and Adolescents” issued by the National Health Commission of China. This industry standard provides BMI reference cut-points for Chinese school-age children (14).

Precocious puberty: Diagnosed by pediatric endocrinologists based on the presence of secondary sexual characteristics (Tanner stage ≥2) before age 8 in girls and age 9 in boys, as outlined in the consensus on pathogenesis, diagnosis, and treatment of central precocious puberty by Latronico AC et al (15). The diagnosis was supported by auxological findings (accelerated growth velocity and/or advanced bone age >2 SD above chronological age) and, when clinically indicated, pubertal response to a GnRH stimulation test.

Age groups were defined as: preschool (3–5 years), primary school (6–11 years), and secondary school (12–14 years). Seasons were defined by the local climate: Spring (March-May), Summer (June-August), Autumn (September-November), and Winter (December-February).

### 2.3 Sample collection and vitamin analysis

Fasting venous blood (3–5 mL) was collected in serum separator tubes. After clotting, samples were centrifuged at 3000 × g for 10 min. Serum was aliquoted and stored at −80 °C until analysis.

Serum concentrations of VA (retinol), VD (25-hydroxyvitamin D3), VE (*α*-tocopherol), VK1, and VK2 (MK-4) were simultaneously quantified using a validated LC-MS/MS method (AB Sciex 5500 system). Briefly, 200 μL of serum was mixed with isotopically labeled internal standards and extracted with n-hexane. The dried extract was reconstituted and injected into a C18 reversed-phase column. Analytes were detected in multiple reaction monitoring (MRM) mode. The method demonstrated good linearity, precision (CV < 10%), and recovery. Deficiency/sufficiency cut-offs were based on established guidelines: VA <0.2 mg/L (deficient), 0.2–0.3 mg/L (marginal) (10); VD <20 ng/mL (16); VE <5 mg/L; VK1 and VK2 < 0.1 ng/mL (17).

### 2.4 Statistical analysis

Statistical analyses were performed using SPSS 26.0 and GraphPad Prism 10. Normality was assessed using the Kolmogorov–Smirnov test. Normally distributed continuous variables are expressed as mean ± standard deviation (SD) and compared using Student's t-test or one-way ANOVA; non-normally distributed variables are expressed as median (interquartile range, IQR) and compared using the Mann–Whitney U test or Kruskal–Wallis test. Categorical variables are presented as counts (%) and compared using the *χ*^2^ test.

For all univariate between-group comparisons (by sex, age group, growth status, and season), the Bonferroni correction was applied to adjust for multiple testing. Given that five fat-soluble vitamins (A, D, E, K1, K2) were evaluated concurrently, the significance threshold was set at α = 0.05/5 = 0.01. Adjusted *P*-values (Padj) are reported for these univariate analyses.

Multivariate linear regression models were constructed to evaluate independent associations between vitamin levels and growth phenotypes. Covariates included age, sex, BMI, and season. Interaction terms between covariates were tested, and those with *P*> 0.1 were excluded from the final models. Regression results are presented with unadjusted *P*-values. A two-sided *P*< 0.05 was considered statistically significant for regression analyses.

## 3 Results

### 3.1 Characteristics of the study population

The study included 1,802 children with a mean age of 8.5 ± 3.2 years. The distribution by growth status was: healthy (*n*=1104), short stature (*n*=236), obesity (*n*=94), and precocious puberty (*n*=368). Demographic details are shown in Table 1.

**Table 1:** Number of diagnoses of different characteristics of children aged 3−14 in ruian city from October 2022 to February 2024.

Age	Number surveyed		Health Group		Short group		Obesity group		Precocious puberty group	
(Years)	Boy	Girl	Boy	Girl	Boy	Girl	Boy	Girl	Boy	Girl
3	97	78	84	76	12	2	1	0	0	0
4	124	84	101	69	23	9	0	1	0	5
5	120	87	83	64	34	13	3	1	0	9
6	116	98	87	56	19	6	7	3	3	33
7	87	122	70	44	13	9	3	2	1	67
8	67	175	56	28	10	15	1	5	0	127
9	75	157	48	28	11	10	11	15	5	104
10	58	45	40	21	6	11	10	2	2	11
11	69	29	46	25	10	2	13	2	0	0
12	46	20	26	16	10	2	9	2	1	0
13	23	7	17	7	5	0	1	0	0	0
14	10	8	5	7	4	0	1	1	0	0
TOTAL	892	910	663	441	157	79	60	34	12	356

### 3.2 Overall vitamin status and prevalence of deficiency

As shown in Table 2, Table 3, VD and VK2 exhibited the highest prevalence of insufficiency. The overall insufficiency rates were: for VD, 13.6% in boys and 22.4% in girls. Specifically, the overall insufficiency rates were: for VD, 13.6% (121/892) in boys and 22.4% (204/910) in girls; for VK2, 36.0% (321/892) in boys and 39.8% (362/910) in girls. Deficiency rates for VA, VE, and VK1 were considerably lower (<10% for both sexes).

**Table 2:** Distribution of fat-soluble vitamin insufficiency and sufficiency in boys by age group [n (%)].

Age (years)	Number	Vitamin A Insufficiency (<0.2 mg/L)	Vitamin D Insufficiency (<20 ng/mL)	Vitamin E Insufficiency (<5 mg/L)	Vitamin K1 Insufficiency (<0.1 ng/mL)	Vitamin K2 Insufficiency (<0.1 ng/mL)
3	97	1 (1.0%)	1 (1.0%)	0 (0%)	7 (7.2%)	43 (44.3%)
4	124	1 (0.8%)	8 (6.5%)	0 (0%)	10 (8.1%)	32 (25.8%)
5	120	1 (0.8%)	7 (5.8%)	1 (0.8%)	9 (7.5%)	41 (34.2%)
6	116	0 (0%)	19 (16.4%)	0 (0%)	9 (7.8%)	36 (31.0%)
7	87	5 (5.7%)	12 (13.8%)	0 (0%)	10 (11.5%)	31 (35.6%)
8	67	1 (1.5%)	6 (9.0%)	0 (0%)	6 (9.0%)	29 (43.3%)
9	75	1 (1.3%)	19 (25.3%)	0 (0%)	6 (8.0%)	35 (46.7%)
10	58	0 (0%)	11 (19.0%)	0 (0%)	4 (6.9%)	23 (39.7%)
11	69	0 (0%)	19 (27.5%)	0 (0%)	11 (15.9%)	19 (27.5%)
12	46	0 (0%)	8 (17.4%)	1 (2.2%)	4 (8.7%)	16 (34.8%)
13	23	0 (0%)	8 (34.8%)	0 (0%)	1 (4.3%)	13 (56.5%)
14	10	0 (0%)	3 (30.0%)	0 (0%)	0 (0%)	3 (30.0%)
Total	892	10 (1.1%)	121 (13.6%)	2 (0.2%)	77 (8.6%)	321 (36.0%)

**Table 3:** Distribution of fat-soluble vitamin insufficiency and sufficiency in girls by age group [n (%)].

Age (years)	Number	Vitamin A Insufficiency (<0.2 mg/L)	Vitamin D Insufficiency (<20 ng/mL)	Vitamin E Insufficiency (<5 mg/L)	Vitamin K1 Insufficiency (<0.1 ng/mL)	Vitamin K2 Insufficiency (<0.1 ng/mL)
3	78	0 (0%)	1 (1.3%)	0 (0%)	2 (2.6%)	22 (28.2%)
4	84	4 (4.8%)	3 (3.6%)	0 (0%)	5 (6.0%)	33 (39.3%)
5	87	2 (2.3%)	7 (8.0%)	0 (0%)	4 (4.6%)	28 (32.2%)
6	98	1 (1.0%)	21 (21.4%)	0 (0%)	13 (13.3%)	33 (33.7%)
7	122	0 (0%)	16 (13.1%)	1 (0.8%)	12 (9.8%)	51 (41.8%)
8	175	2 (1.1%)	46 (26.3%)	0 (0%)	27 (15.4%)	86 (49.1%)
9	157	0 (0%)	51 (32.5%)	0 (0%)	12 (7.6%)	66 (42.0%)
10	45	0 (0%)	20 (44.4%)	0 (0%)	6 (13.3%)	18 (40.0%)
11	29	0 (0%)	17 (58.6%)	0 (0%)	2 (6.9%)	8 (27.6%)
12	20	0 (0%)	13 (65.0%)	0 (0%)	2 (10.0%)	10 (50.0%)
13	7	0 (0%)	4 (57.1%)	0 (0%)	3 (42.9%)	2 (28.6%)
14	8	0 (0%)	5 (62.5%)	0 (0%)	3 (37.5%)	4 (50.0%)
Total	910	9 (1.0%)	204 (22.4%)	1 (0.1%)	91 (10.0%)	362 (39.8%)

### 3.3 Variations by sex, age, and season

Sex: Three-year-old girls had significantly higher VA than boys (0.39 ± 0.08 vs. 0.36 ± 0.07 mg/L, adjusted *P* < 0.01). Boys had higher VD levels than girls after age 10, with the most significant difference at age 12 (23.97 ± 3.94 vs. 16.04 ± 6.22 ng/mL, adjusted *P* < 0.0001).

Age: A significant downward trend in VD levels with increasing age was observed in both sexes (adjusted *P* < 0.0001).

Season: Pronounced seasonal patterns of vitamin insufficiency were identified (Tables 4–6).

**Table 4:** seasonal patterns of vitamin A status (serum retinol) in children with marginal deficiency.

Season	Age (years)	Sex	Vitamin A Level (mg/L, Mean ± SD)
Spring	11	Girls	0.27 ± 0.07

Marginal deficiency for vitamin A is defined as serum retinol concentration between 0.20 and 0.30 mg/L. Only groups identified as having marginal deficiency or deficiency in specific seasons are listed.

**Table 5:** Seasonal patterns of vitamin D deficiency [Serum 25(OH)D <20 ng/mL] in Children.

Season	Age (years)	Sex	Vitamin D Level (ng/mL, Mean ± SD)
Spring	10	Girls	18.15 ± 6.33
Spring	11	Girls	12.85 ± 1.77
Summer	11	Girls	19.98 ± 4.54
Summer	12	Girls	16.97 ± 5.81
Summer	13	Girls	16.66 ± 4.63
Winter	9	Girls	19.82 ± 6.05
Winter	11	Girls	18.98 ± 6.54
Winter	12	Girls	16.35 ± 10.05
Winter	13	Boys	19.72 ± 1.25

Deficiency for vitamin D is defined as serum 25-hydroxyvitamin D concentration <20 ng/mL. Only groups identified as deficient in specific seasons are listed.

**Table 6:** seasonal patterns of vitamin K2 deficiency (serum MK-4 < 0.10 ng/mL) in children.

Season	Age (years)	Sex	Vitamin K2 Level (ng/mL, Mean ± SD)
Spring	8	Boys	0.09 ± 0.06
Spring	9	Boys	0.09 ± 0.03

Deficiency for vitamin K2 is defined as serum menaquinone-4 (MK-4) concentration <0.10 ng/mL. only groups identified as deficient in specific seasons are listed.

### 3.4 Associations with growth and development status

**Figure 1:**
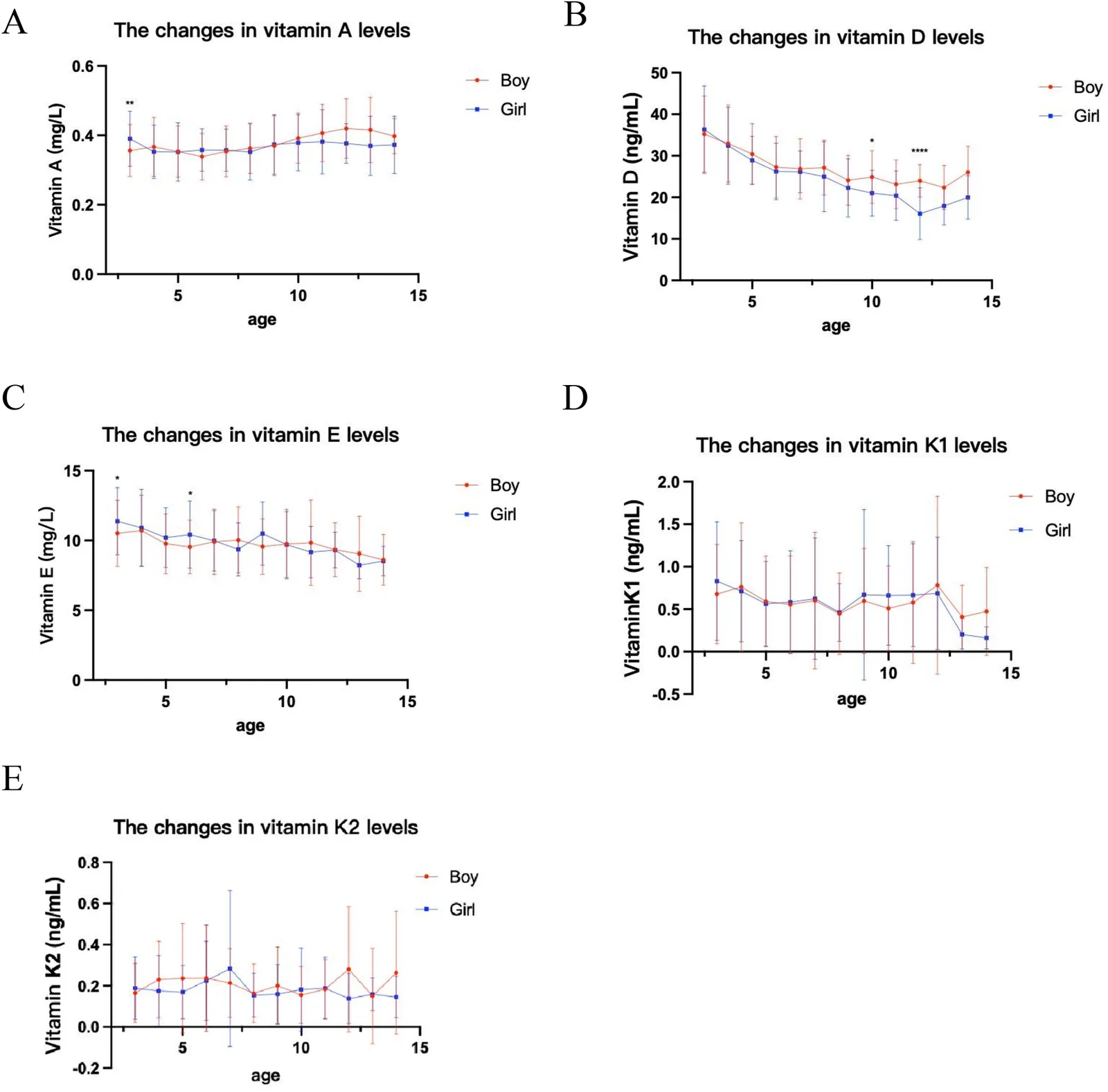
Age- and sex-specific trajectories of serum fat-soluble vitamin levels in children. Serum concentrations of **(A)** Vitamin A, **(B)** Vitamin D, **(C)** Vitamin E, **(D)** Vitamin K1, and **(E)** Vitamin K2 are plotted against age for boys (blue circles/solid line) and girls (red squares/dashed line). Data are presented as mean ± SD. Asterisks indicate statistically significant differences between boys and girls at specific age points after Bonferroni correction (*P* < 0.05, \\*P* < 0.01, *P* < 0.001).

**Figure 2:**
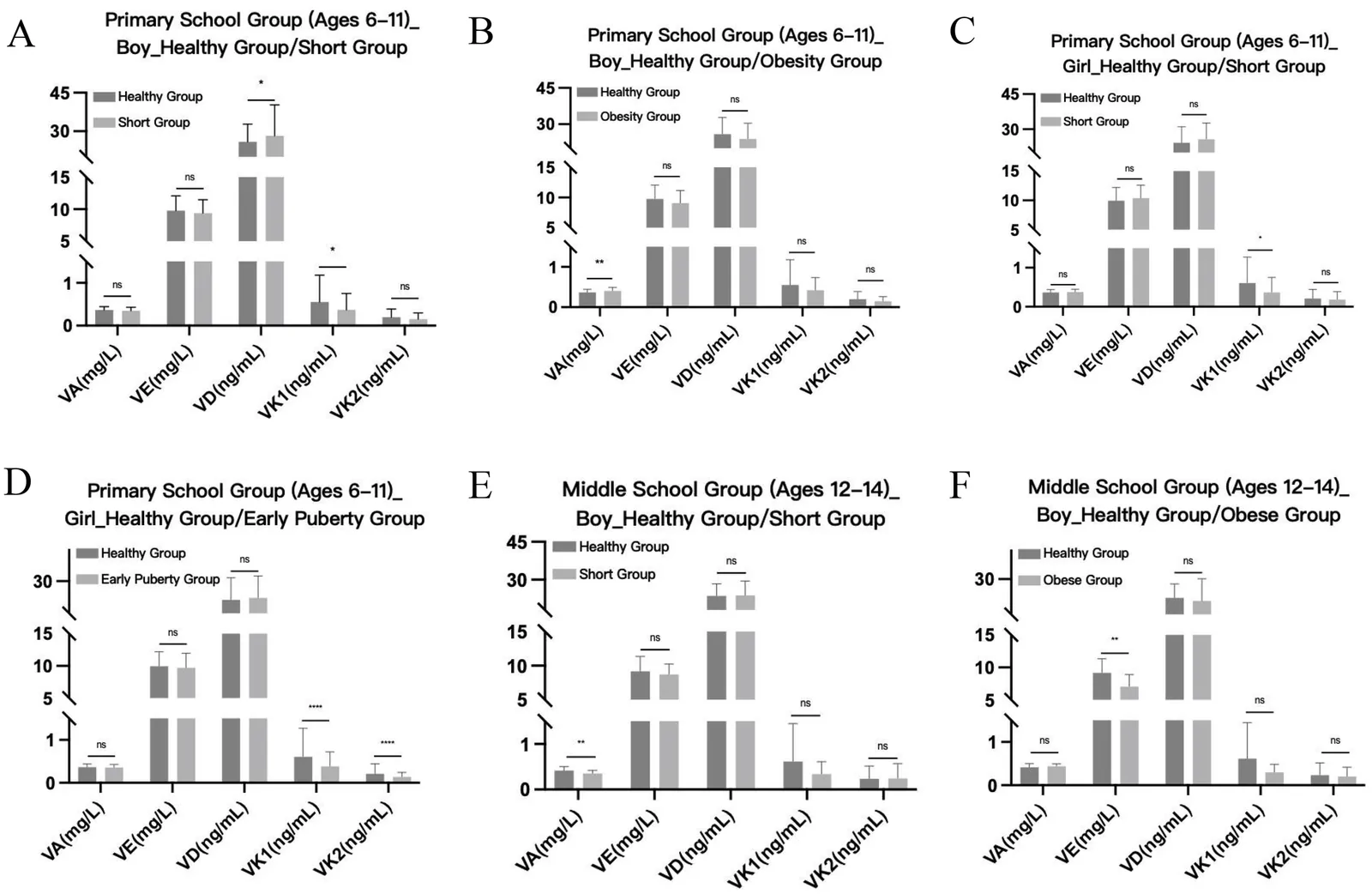
Comparison of serum fat-soluble vitamin levels among children with different growth and developmental statuses. Box-and-whisker plots show median, interquartile range, and 5th-95th percentile. **(A)** Serum VA, VE, VD, VK1, VK2 in primary school boys (ages 6−11): healthy (*n*=366) vs. Short stature (*n*=79). **(B)** Serum VA, VE, VD, VK1, VK2 in primary school boys (Ages 6−11): Healthy (*n*=366) vs. Obesity (*n*=60). **(C)** Serum VA, VE, VD, VK1, VK2 in primary school girls (Ages 6−11): Healthy (*n*=201) vs. Short stature (*n*=34) vs. Precocious puberty (*n*=356). **(D)** Serum VA, VE, VD, VK1, VK2 in primary school girls (Ages 6−11): Healthy (*n*=201) vs. Precocious puberty (*n*=356). **(E)** Serum VA, VE, VD, VK1, VK2 in middle school boys (Ages 12−14): Healthy (*n*=96) vs. Short stature (*n*=12). **(F)** Serum VA, VE, VD, VK1, VK2 in middle school boys (Ages 12−14): Healthy (*n*=96) vs. Obesity (*n*=12). *P*-values are from Mann–Whitney U or Kruskal–Wallis tests with Bonferroni correction applied for the number of comparisons within each vitamin/age-group panel (*P* < 0.05, \\*P* < 0.01, *P* < 0.001, \\\\*P* < 0.0001). Only significant comparisons are shown.

Univariate analyses revealed notable differences between the healthy group and other phenotypes (Figures 1, 2).

#### 3.4.1 Univariate comparisons

In primary school boys, VD levels were lower in the healthy group than in the short stature group (*P* < 0.05).

Healthy boys had lower VA levels than obese boys (*P* < 0.01).

Most strikingly, in primary school girls, both VK1 and VK2 levels were significantly higher in the healthy group compared to the precocious puberty group (*P* < 0.0001).

#### 3.4.2 Multivariate regression analysis

Multivariate linear regression, adjusting for age, sex, and season, was performed to assess the independent associations.

The higher VD levels observed in short-stature boys compared to healthy boys in univariate analysis did not remain statistically significant after adjusting for confounders (*β* = −0.15, *P* = 0.23).

The higher VA levels in obese boys compared to healthy boys remained statistically significant after adjustment (*β* = 0.04, *P* = 0.03).

Crucially, the inverse association between VK2 levels and precocious puberty in girls was robust to adjustment for age, BMI, and season (β = −0.08, *P* < 0.001). Similarly, VK1 levels were significantly lower in the precocious puberty group (β = −0.11, *P* = 0.004).

## 4 Discussion

The study provides a comprehensive assessment of five fat-soluble vitamins in a large pediatric cohort and reveals significant associations with growth disorders. Our key finding—the independent, inverse association between circulating VK (both K1 and K2) and precocious puberty in girls—suggests a previously underappreciated link that merits further investigation.

The high prevalence of VD and VK2 deficiency aligns with and extends findings from recent national surveys (9) and a recent study on VK status in Chinese children (8). The observed seasonal patterns—VD deficiency peaking in winter/spring and VK2 deficiency in spring among boys—are consistent with known influences of sunlight exposure and potentially seasonal food availability. The higher VD deficiency rate in older girls may be compounded by lifestyle factors such as reduced outdoor activity and sunscreen use. These findings underscore the need for public health strategies promoting seasonal, vitamin-specific supplementation.

The observation of lower VK levels in girls with precocious puberty presents a novel and biologically plausible hypothesis.

From a physiological standpoint, Vitamin K, particularly VK2, serves as an essential cofactor for the *γ*-carboxylation of osteocalcin, a bone matrix protein secreted by osteoblasts during bone formation (18). Given that precocious puberty involves accelerated linear growth and bone maturation (19), it is hypothesized that increased bone turnover may elevate the demand for VK, potentially resulting in a state of relative depletion or increased utilization of circulating VK (12). This concept aligns with studies showing that the undercarboxylated form of osteocalcin (a marker of low VK status) increases during phases of rapid bone growth. Furthermore, a potential synergy exists between VD and VK: adequate VD promotes osteocalcin synthesis, but without sufficient VK, this protein remains undercarboxylated and functionally inactive (12). However, it is crucial to emphasize that our cross-sectional design fundamentally precludes any causal inference. We cannot determine whether lower VK levels are a consequence of rapid growth and increased bone metabolism in precocious puberty, or whether they represent a pre-existing condition that might contribute to the disorder's pathophysiology. The potential influence of elevated sex hormones on VK metabolism also warrants investigation. Therefore, this finding should be regarded strictly as exploratory and hypothesis-generating. It identifies a significant statistical association that necessitates further verification through prospective cohort studies and ultimately, elucidation through mechanistic laboratory research.

The finding of higher VA levels in boys with obesity is consistent with the hypothesis of sequestration in expanded adipose tissue or volumetric dilution, as VA is a fat-soluble vitamin (20). Regarding the initially observed higher VD levels in short-stature boys, this association was not robust in multivariate analysis. We hypothesize this may be due to unmeasured confounding, such as clinical intervention. For instance, parents of children perceived as “short” may be more likely to provide vitamin supplements, including VD. This interpretation remains speculative and should be considered a research hypothesis, as our study did not collect data on vitamin supplementation history, dietary intake, or other potential confounders that could have influenced these findings.

## 5 Strengths and limitations

This study has several strengths, including its large sample size, simultaneous quantification of five fat-soluble vitamins using the gold-standard LC-MS/MS method, and detailed clinical phenotyping combined with multivariate statistical adjustments.

However, several limitations must be acknowledged. First, the cross-sectional design prevents establishment of causal or temporal relationships. Second, the single-center setting in Eastern China may limit the generalizability of findings to other populations with different dietary habits and climates. Third, and most significantly, we lacked data on key potential confounders including detailed dietary intake, sunlight exposure duration, physical activity levels, socioeconomic status, and vitamin supplementation history. The absence of these data precludes a more definitive analysis of the independent effects of growth phenotypes on vitamin status and may introduce residual confounding. Fourth, the severe gender imbalance in the precocious puberty group (predominantly girls) limits the generalizability of our VK findings to boys with this condition. Finally, while LC-MS/MS provides accurate measurements, a single measurement may not fully capture long-term vitamin status.

## 6 Conclusion

We documented a high burden of VD and K2 deficiency among children in Eastern China, with clear demographic and seasonal patterns. In multivariate analysis, a significant, independent inverse association was found between VK status and precocious puberty in girls. This novel finding is hypothesis-generating and warrants further mechanistic and longitudinal investigation to determine its clinical significance and the direction of causality. From a clinical perspective, our results support the need to move beyond a “one-size-fits-all” approach to micronutrient assessment. Clinicians should be aware of the high prevalence of VD and VK2 insufficiency in this population and consider targeted, seasonally adapted nutritional counseling or supplementation, particularly for adolescent girls and children with accelerated growth.

## Data Availability

The original contributions presented in the study are included in the article/Supplementary Material, further inquiries can be directed to the corresponding authors.
